# Supplementary figures and images for: New role for an old acquaintance: miR-1246 as a new inflammatory and prognostic marker in polytrauma patients
Source: PeerJ. 2025 Apr 7;13:e19185. doi: 10.7717/peerj.19185 (PMC11984479; doi:10.7717/peerj.19185)

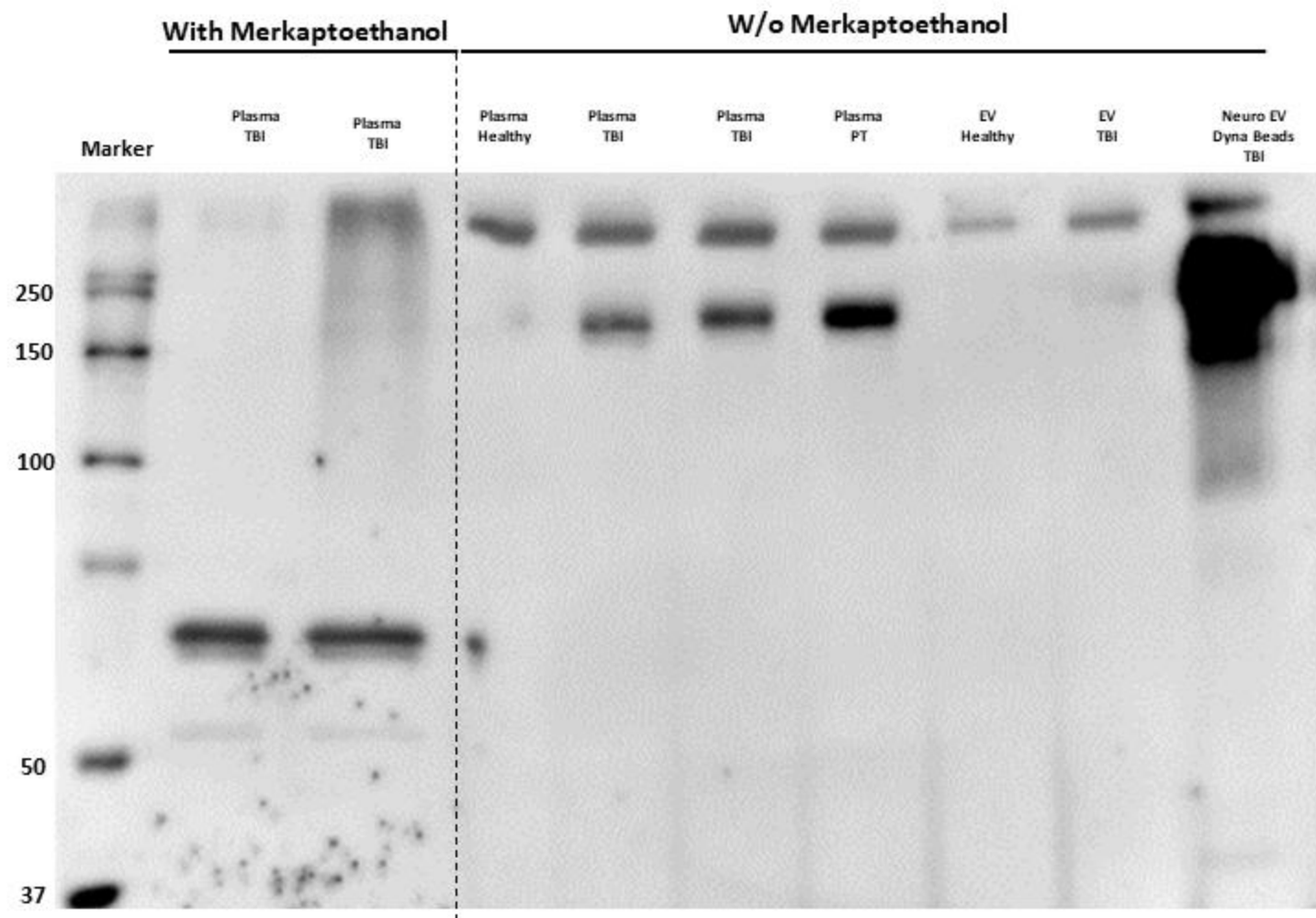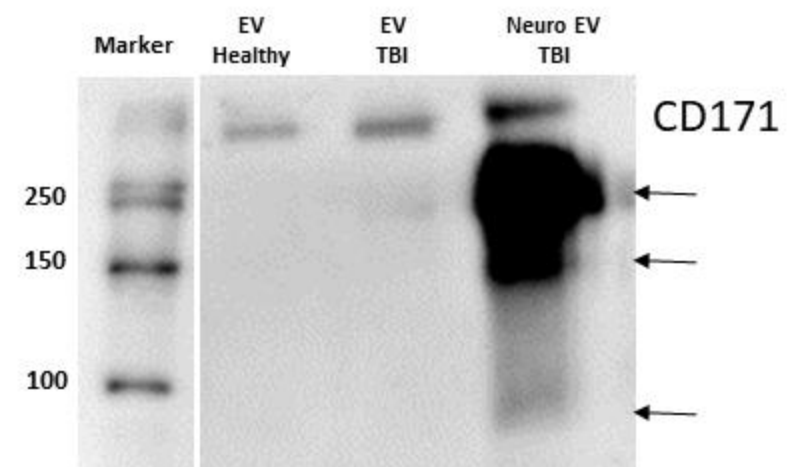

AB: CD171 (REF:20659-1-AP) 1:1000

~ 200-220 kDa

~ 140 kDa

~ 80 kDa

Gel: 7%

Supplement: Supplemental Information 5 — To validate the isolation of neuro (CD171+) EVs, enrichment of EV epitope was analyzed on EV samples (20μg protein equivalent) by means of western blot. The gel separation was performed under non-reducing conditions. Antibodies against CD171 (Proteintech, 20659-1-AP, 1:1000) and anti-rabbit IgG, horseradish peroxidase (HRP)-linked antibody (Cell signaling Technology, #7074,1:2000) were used for detection. Expected CD171 protein sizes 200-220 kDa, 140 kDa and 80 kDa. [file peerj-13-19185-s005.pdf]
